# Osimertinib combined with bevacizumab for leptomeningeal metastasis from *EGFR*‐mutation non‐small cell lung cancer: A phase II single‐arm prospective clinical trial

**DOI:** 10.1111/1759-7714.13738

**Published:** 2020-11-17

**Authors:** Zhi‐qin Lu, Jing Cai, Xia Wang, Jian‐ping Wei, Zhi‐min Zeng, Long Huang, An‐wen Liu

**Affiliations:** ^1^ Department of Oncology The Second Affiliated Hospital of Nanchang University Nanchang China; ^2^ Department of Oncology Jiangxi Key Laboratory of Clinical Translational Cancer Research Nanchang China

**Keywords:** Bevacizumab, *EGFR* mutation, leptomeningeal metastasis, non‐small cell lung cancer, osimertinib

## Abstract

**Background:**

Leptomeningeal metastasis (LM) is associated with poor prognosis in non‐small cell lung cancer (NSCLC). The aim of this study was to investigate the efficacy and safety of osimertinib combined with bevacizumab for LM from epidermal growth factor receptor mutation (EGFRm) NSCLC.

**Methods:**

We conducted a phase II single‐arm prospective clinical trial of EGFRm NSCLC with LM treated with osimertinib combined with bevacizumab. LM response assessment was based on the modified RANO LM radiological criteria; CNS and extra‐CNS response was evaluated according to the Response Evaluation Criteria in Solid Tumors (RECIST) version 1.1. The primary end points included LM progression‐free survival (PFS) and objective response rate (ORR); the secondary end points included safety and LM overall survival (OS).

**Results:**

A total of 14 patients were included in the study, with a median age of 61 years, and they were predominantly female (64%). *EGFR* mutations were reported in exons 19 del (*n* = 7) and 21 L858R (*n* = 7). When LM was diagnosed, 12 (85.7%) patients had clinical symptoms, 71.4% (10/14) of patients were diagnosed with LM by cytology, and five (35.7%) patients had a performance status (PS) score > 2. The median LM PFS was 9.3 months (95% CI: 8.2–10.4), and the LM ORR was 50%. The safety findings in the present study were consistent with the known profile of osimertinib with bevacizumab; the median LM OS was 12.6 months, and the one‐year survival rate was 35.7%.

**Conclusions:**

Osimertinib combined with bevacizumab is an appropriate treatment option for patients with LM from EGFRm NSCLC.

**Key points:**

**Significant findings of the study:**

To date, there is no prospective clinical study on the treatment of osimertinib combined with bevacizumab in EGFRm NSCLC with LM.

**What this study adds:**

The median LM PFS was 9.3 months (95% CI: 8.2–10.4), and the LM ORR was 50%, the median LM OS was 12.6 months, and the one‐year survival rate was 35.7%. Osimertinib combined with bevacizumab is an appropriate treatment option for patients with LM from EGFRm NSCLC.

## Graphical abstract

we evaluated the first phase II prospective clinical trial to assess the efficacy and safety of osimertinib combined with bevacizumab for LM from EGFRm NSCLC. Osimertinib combined with bevacizumab is an appropriate treatment option for patients with LM from EGFRm NSCLC.

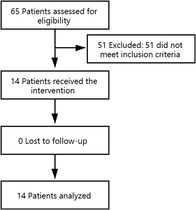

## Introduction

Lung cancer remains a leading cause of death worldwide, and most cases of non‐small cell lung cancer (NSCLC) are diagnosed at an advanced stage.^1^ Leptomeningeal metastasis (LM) is a fatal complication of advanced NSCLC associated with poor prognosis and rapid deterioration of performance status. ^2^, ^3^ The incidence of LM is increasing, reaching 3.8% in molecularly unselected NSCLC patients, being more frequent in the adenocarcinoma subtype and occurring in up to 9.4% in epidermal growth factor receptor mutation (EGFRm) lung cancer patients; one‐third of patients have concomitant brain metastasis. ^4^, ^5^, ^6^, ^7^ This increased incidence may in part be conducive to the increased survival of patients with EGFRm advanced NSCLC since the introduction of EGFR‐tyrosine kinase inhibitors (TKIs).^8^

Currently, no standard therapeutic regimen for LM with EGFRm NSCLC has been established because of its rarity and heterogeneity.^9^ TKIs are the first‐line treatment of choice for patients with EGFRm NSCLC. The leptomeningeal space is a sanctuary site for tumor cells and therapeutic agents due to the presence of an active blood‐brain barrier (BBB).^10^ Therefore, CSF concentration is an important factor affecting the treatment of LM with TKIs.^9^, ^11^, ^12^ Standard dose first‐ and second‐generation EGFR‐TKIs have good systemic efficacy but suboptimal central nervous system (CNS) penetration, as evidenced by preclinical studies of brain distribution and clinical reports of CSF penetration.^13^ Osimertinib is a third‐generation irreversible, oral EGFR‐TKI that potently and selectively inhibits both EGFR‐TKI sensitizing and EGFR T790M resistance mutations that has demonstrated efficacy in NSCLC CNS metastasis.^14^, ^15^, ^16^, ^17^, ^18^, ^19^ Preclinical, phase I/II clinical studies and the AURA program (AURA extension, AURA2, AURA17 and AURA3) have shown that osimertinib has higher brain permeability than first‐ and second‐generation treatment.^13^, ^20^

Bevacizumab is a recombinant humanized monoclonal antibody against vascular endothelial growth factor (VEGF), and animal studies and autopsy specimens have shown that VEGF plays an important role in LM.^21^ VEGF and EGFR share many overlapping and parallel downstream pathways.^22^ Biological rationale shows that the inhibition of the EGFR and VEGR signaling pathways could improve the efficacy of antitumor therapy and remove the resistance of EGFR inhibition.^23^, ^24^ Osimertinib and bevacizumab both cross the BBB and have comparable effectiveness in the CNS.^18^ In addition, preclinical studies have shown similar results.^25^ Based on these findings, a number of clinical trials have confirmed that VEGF inhibitors in combination with EGFR‐TKIs significantly prolong patient survival. To date, there is no prospective clinical study on the treatment of osimertinib combined with bevacizumab in EGFRm NSCLC with LM, and this needs to be further studied. Therefore, we conducted a phase II prospective study to evaluate the efficacy and safety of osimertinib combined with bevacizumab for EGFRm NSCLC with LM (NCT04425681) to seek effective methods for the treatment of such patients.

## Methods

### Patient selection

The eligibility criteria were as follows: (i) age 18–80 years; (ii) histologically or cytologically confirmed NSCLC; (iii) the detection of an *EGFR* mutation, with EGFR status identified from primary lung tumors using the amplification refractory mutation system (ARMS) or next‐generation sequencing (NGS) analysis; (iv) LM defined by CSF positivity for malignant cells and/or focal or diffuse enhancement of leptomeninges, nerve roots or the ependymal surface diagnosed by magnetic resonance imaging (MRI) with gadolinium contrast; (v) adequate organ function; and (vi) signed informed consent form.

The exclusion criteria were: (i) patients with clinical manifestations of nervous system failure, including severe encephalopathy, grade III–IV white matter lesions confirmed by imaging examination, moderate or severe coma, or a Glasgow Coma Score less than nine points; (ii) allergy to osimertinib or bevacizumab; (iii) pregnant women, nursing women, or men or women of childbearing potential who were unwilling to employ adequate contraception; (iv) a history of myocardial infarction or other evidence of arterial thrombotic disease (angina) or symptomatic congestive heart failure (New York Heart Association ≥ grade 2), unstable angina pectoris, or cardiac arrhythmia; note: allowed only if patient had no evidence of active disease for at least six months prior to the study); (v) a history of cerebral vascular accident (CVA) or transient ischemic attack (TIA) ≤ 6 months prior to the study; (vi) a history of bleeding diathesis or coagulopathy; (vii) a history of hemoptysis ≥ grade 2 (defined as bright red blood of at least 2.5 mL) ≤3 months prior to the study; (viii) leukocytes below 2 × 10^9^/L, neutrophils below 1 × 10^9^/L, or platelets below 50 × 10^9^/L; (ix) had major surgery within the past 60 days; (x) a history of arteriovenous thrombosis; (xi) gastrointestinal perforation in the past six months; (xii) inadequately controlled hypertension (systolic blood pressure of >150 mmHg or diastolic pressure > 100 mmHg on antihypertensive medications; note: patients with a history of hypertensive crisis or hypertensive encephalopathy were not allowed); and (xiii) grade 4 proteinuria.

### Treatment

Enrolled patients received osimertinib 80 mg orally daily and bevacizumab 7.5 mg/kg intravenously every three weeks. Treatment continued until the disease progressed, unacceptable adverse events occurred, or the patient withdrew consent.

### Evaluation of efficacy and safety

Four weeks after the initiation of osimertinib and bevacizumab, neurological evaluations, brain magnetic resonance imaging (MRI) and chest/abdominal computed tomography were routinely performed, and were then performed every 1–3 months. LM response assessment was based on the modified RANO LM radiological criteria; CNS and extra‐CNS response was evaluated according to the Response Evaluation Criteria in Solid Tumors (RECIST) version 1.1. LM progression‐free survival (LM PFS) was calculated as the time from LM diagnosis to the first documentation of disease progression or death. The objective response rate (ORR) was defined as the proportion of patients with the best overall response for complete response and partial response (CR + PR). The disease control rate (DCR) was calculated as the proportion of patients with an objective response or stable disease for at least four weeks. Overall survival of LM (LM OS) was defined as the period from diagnosis of LM to death or last follow‐up. OS status was classified as censored if a patient was unavailable for follow‐up or survived beyond the last follow‐up (1 June 2020). Overall survival (OS) was calculated from the first diagnosis of NSCLC until the date of death or when the patients were censored at the last follow‐up. Adverse events (AEs) were evaluated based on the National Cancer Institute Common Toxicity Criteria, version 4.0. Dose‐limiting toxic effects included grade 3 or higher hemoptysis or epistaxis, other grade 4 hematological toxic effects, grade 4 or higher creatinine elevation, proteinuria or diarrhea, any other nonhematological grade 3 or higher major organ toxic effects, or death associated with the regimen.

The primary end points included LM PFS and ORR; the secondary end points included safety and LM OS.

### Statistical analysis

To consider combined osimertinib and bevacizumab as a promising treatment strategy, we planned to detect an improvement in PFS from three months to 9.0 months with approximately 80% power using a stratified log‐rank test at a one‐sided significance level of 0.20, assuming a 10% exclusion rate. The planned sample size was 14 patients. Survival analyses were performed according to the Kaplan‐Meier method, and confidence intervals (CIs) were calculated at a 95% confidence level. All analyses were performed using IBM SPSS software, version 24.0, and a diagram was made using GraphPad Prism software version 8.0.

### Ethics

This study adhered to the rules and regulations of clinical studies with respect to human subject protection, and it was approved by the Second Affiliated Hospital of Nanchang University Medical Ethics Committee and complied with the Declaration of Helsinki. Informed consent was obtained from all enrolled patients. The trial is registered with ClinicalTrials.gov (NCT04425681).

## Results

### Patient characteristics

**Figure 1:**
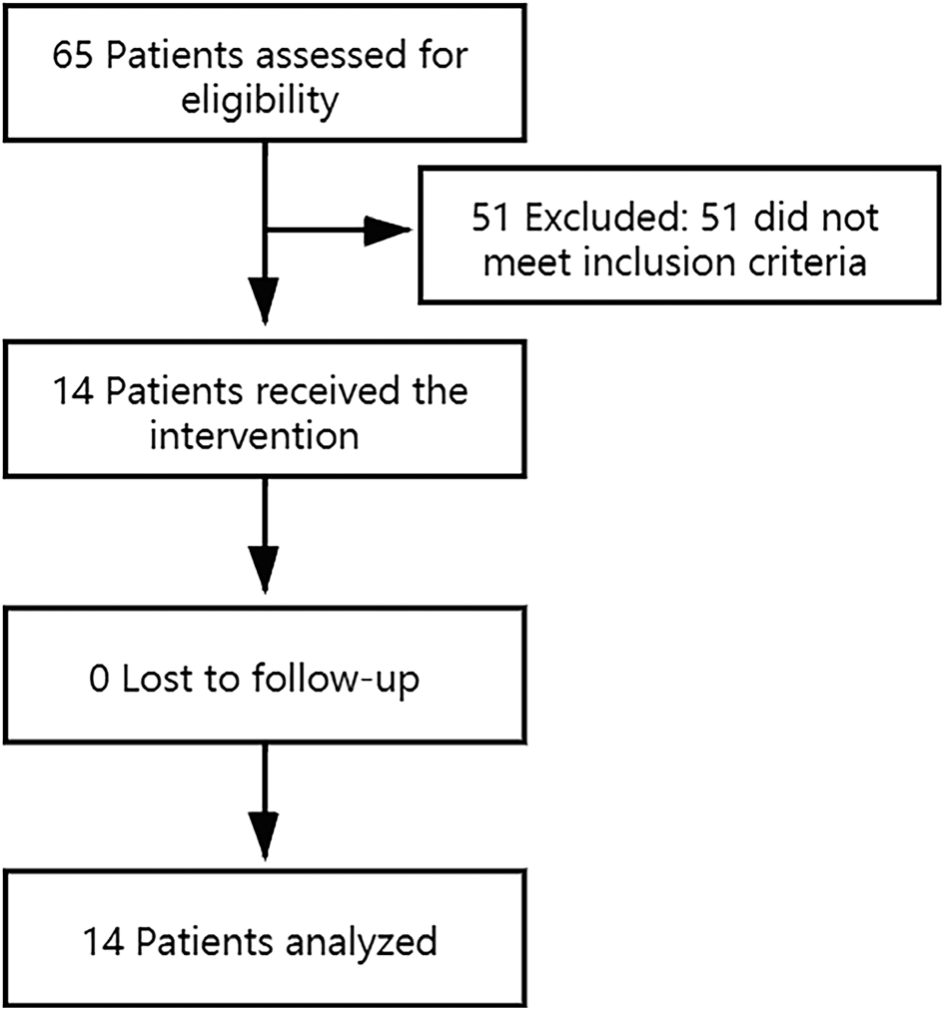
Consort diagram.

A total of 14 patients, including five men and nine women, were enrolled into this study from October 2017 to August 2019 at the Second Affiliated Hospital of Nanchang University (Fig 1). The patient characteristics are listed in Table 1. The median age was 61 (range 50–75). All tumor histological analyses indicated adenocarcinoma. *EGFR* mutations at diagnosis were located in exons 19 del (*n* = 7) and 21 L858R (*n* = 7), and none had the T790M mutation. With NSCLC diagnosis, LM was reported in 28.6% (4/14) of patients, and for initial TKI treatment, seven patients were treated with gefitinib, one patient was treated with erlotinib, and one patient was treated with icotinib. A total of 85.7% (12/14) of patients had LM‐related clinical symptoms, 71.4% (10/14) of patients were diagnosed with LM by cytology, and 42.9% (6/14) were diagnosed by MRI scan and CSF cytopathology. Performance status was >2 for 35.7% (5/14) of patients.There were 11 patients who had concomitant brain metastasis, and 35.7% (5/14) of patients had received brain radiotherapy. Two of the patients received whole‐brain radiotherapy (WBRT), one patient received SRS for brain parenchymal metastasis, one patient received whole‐brain whole‐neck medulla radiotherapy, and one patient received simultaneous modulated accelerated radiation therapy for the brain (SMART‐Brain).

**Table 1:** Patient characteristics

Characteristics	No (%)
Total case	14
Age (years)	
Median	61
Range	50–75
Gender	
Male	5 (36)
Female	9 (64)
Histology	
Adenocarcinoma	14 (100.0)
*EGFR* mutation	
Exon 19 del	7 (50.0)
Exon 21 L858R	7 (50.0)
Initial diagnosis was LM	
Yes	4 (28.6)
No	10 (71.4)
Initial TKI treatment	
Gefitinib	7 (50)
Erlotinib	1 (7.1)
Icotinib	1 (7.1)
Others	1 (7.1)
Neurological symptoms	
Yes	12 (85.7)
No	2 (14.3)
ECOG performance status score	
≤2	9 (64.3)
>2	5 (35.7)
Concomitant BM	
Yes	11 (78.6)
No	3 (21.4)
Brain radiotherapy	
Yes	5 (35.7)
No	9 (64.3)
CSF cytopathology	
YES	10 (71.4)
Elevated CSF protein	6 (60)
Diagnosis of LM by MRI scan	
Yes	11 (78.6)
Diagnosis of LM by MRI scan and CSF cytopathology	
Yes	6 (42.9)

BM, brain metastasis; CSF, cerebrospinal fluid; ECOG, Eastern Cooperative Oncology Group; EGFR, epidermal growth factor receptor; LM, leptomeningeal metastasis; MRI, magnetic resonance; TKIs, tyrosine kinase inhibitors.

### Clinical responses

At the time of the analysis of *EGFR*‐mutation NSCLC patients with LM, eight patients (57.1%) had died, and the median follow‐up time was 9.9 months. The median time from the date of initial diagnosis with lung cancer to the occurrence of LM was 13.6 months. Of the 14 total patients, 92.9% (13) had a clinical response. The confirmed LM ORR was 50% (7/14); seven of the patients had a PR (50%; Table 2).

**Table 2:** Response to osimertinib and bevacizumab in 14 patients with leptomeningeal metastasis (LM)

Patient	Clinical	Image	CSF	Response determination
1	Stable	Improved	Not done	Response
2	Improved	Stable	Not done	Stable
3	Improved	Improved	Not done	Response
4	Improved	Stable	Not done	Stable
5	Improved	Improved	Not done	Response
6	Improved	Stable	Not done	Stable
7	Improved	Stable	Not done	Stable
8	Improved	Stable	Not done	Stable
9	Improved	Improved	Not done	Response
10	Worse	Worse	Not done	Progression
11	Improved	Stable	Not done	Stable
12	Improved	Improved	Not done	Response
13	Stable	Improved	Not done	Response
14	Improved	Improved	Not done	Response

**Figure 2:**
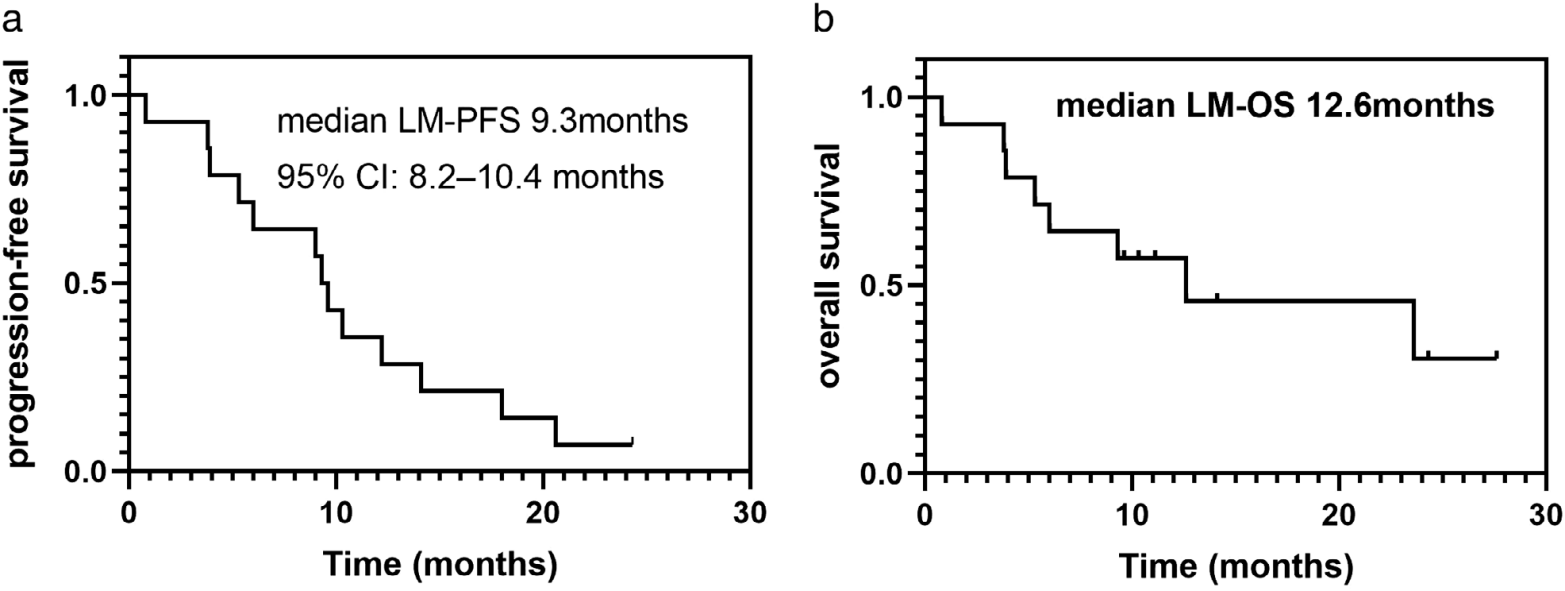
Kaplan‐Meier curve for leptomeningeal metastasis. (**a**) Progression‐free survival (LM‐PFS); and (**b**) overall survival (LM‐OS).

**Figure 3:**
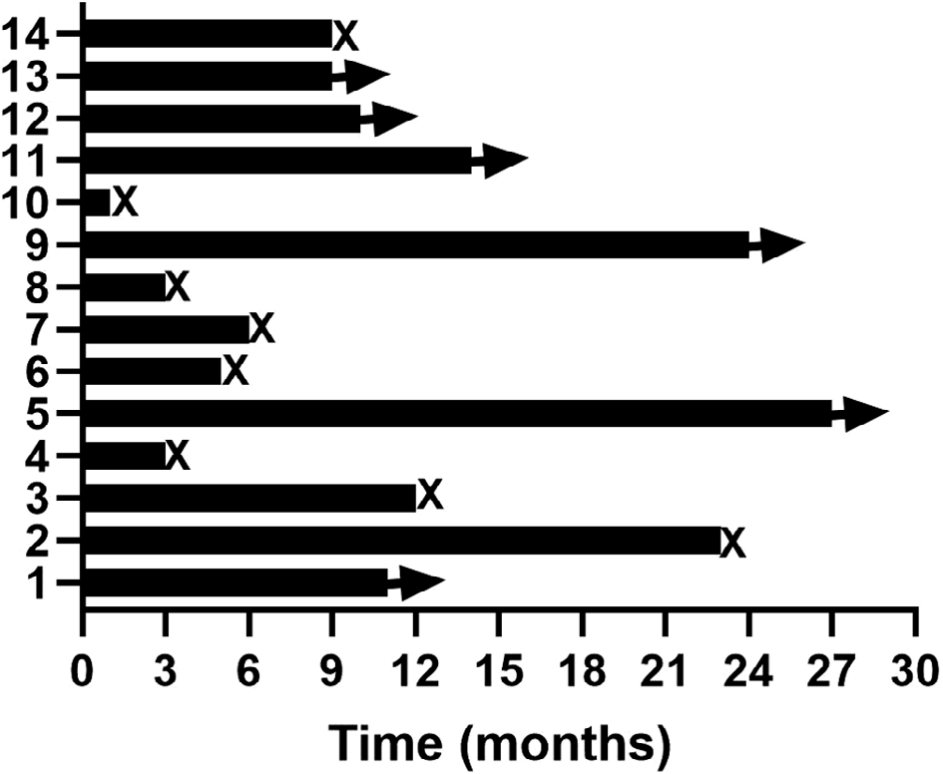
Swimming plot of patients with leptomeningeal metastasis. 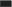 patient, 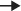 alive, 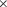 dead.

**Figure 4:**
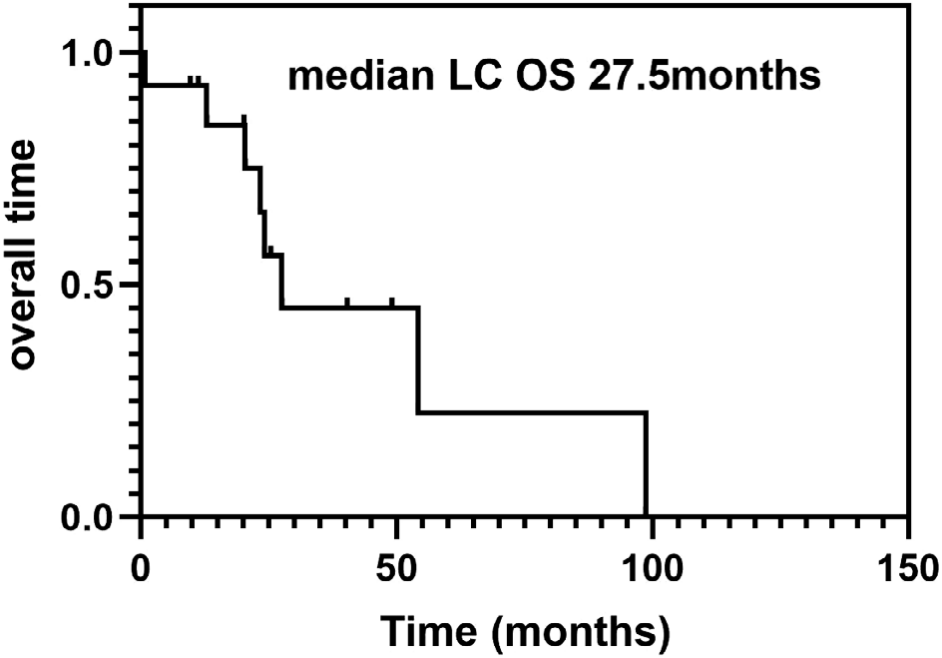
Kaplan‐Meier curve for lung cancer overall survival (LC‐OS).

The primary end point LM PFS was 92.9%. The patients had a median PFS duration of 9.3 months (95% CI: 8.2–10.4) (Fig 2a), median LM OS was 12.6 months (95% CI: 9.8–21.2) (Figs 2, 3), one‐year survival rate was 35.7%, and median lung cancer OS was 27.5 months (Fig 4).

### Safety

The AEs experienced by patients during osimertinib and bevacizumab treatment are listed in Table 3. Leucopenia, thrombocytopenia, anemia, rash, anorexia, fatigue, paronychia, hemoptysis/epistaxis, creatinine elevation, hypertension and proteinuria were frequently reported; the incidence of grade 3 adverse events was low; and no grade 4 or 5 events were observed.

**Table 3:** Adverse events (AEs) graded according to the common toxicity criteria for adverse events (CTCAE)

AEs	ALL	Grade1–2 (%)	Grade 3 (%)	Grade 4 (%)
Hematological				
Anemia	2 (14.3)	2 (14.3)	0 (0)	0 (0)
Thrombocytopenia	3 (21.4)	3 (21.4)	0 (0)	0 (0)
Leucopenia	8 (57.1)	8 (57.1)	0 (0)	0 (0)
Neutropenia	1 (7.1)	1 (7.1)	0 (0)	0 (0)
Nonhematological				
Rash	3 (21.4)	3 (21.4)	0 (0)	0 (0)
Paronychia	2 (14.3)	2 (14.3)	0 (0)	0 (0)
Anorexia	4 (28.6)	4 (28.6)	0 (0)	0 (0)
Fatigue	4 (28.6)	4 (28.6)	0 (0)	0 (0)
Diarrhea	1 (7.1)	1 (7.1)	0 (0)	0 (0)
Interstitial lung disease	0 (0)	0 (0)	0 (0)	0 (0)
Creatinine elevation	3 (21.4)	2 (14.3)	1 (7.1)	0 (0)
Proteinuria	3 (21.4)	3 (21.4)	0 (0)	0 (0)
Hypertension	2 (14.3)	0 (0)	2 (14.3)	0 (0)
Pulmonary embolism	0 (0)	0 (0)	0 (0)	0 (0)
ALT/AST	2 (14.3)	2 (14.3)	0 (0)	0 (0)
Hemoptysis/epistaxis	3 (21.4)	3 (21.4)	0 (0)	0 (0)

## Discussion

To the best of our knowledge, this is the first phase II prospective clinical trial to assess the efficacy and safety of osimertinib combined with bevacizumab for the treatment of EGFRm NSCLC with LM. Osimertinib combined with bevacizumab demonstrated favorable efficacy and safety outcomes in patients with LM from EGFRm NSCLC. The median LM PFS was 9.3 months (95% CI: 8.2–10.4), and the LM ORR was 50%. The safety findings in the present study were consistent with the known profile of osimertinib with bevacizumab. The median LM OS was 12.6 months, and the one‐year LM survival rate was 35.7%.

LM is a fatal complication of advanced NSCLC. LM is different from brain parenchymal metastasis and has a worse prognosis and more severe symptoms. The median survival time after LM treatment is 3–6 months.^6^, ^9^, ^26^, ^27^ Currently, the treatment for LM consists of EGFR‐TKIs, WBRT, systemic/intrathecal chemotherapy, ventriculoperitoneal shunt (VP shunt) and immune checkpoint inhibitors (ICIs).^6^, ^28^, ^29^ However, the survival benefits of these treatments remain poorly established. The poor permeability of chemotherapeutic or targeted agents through the BBB may account for the limited role of these treatments. Although WBRT is an effective treatment for patients with brain metastasis (BM), its therapeutic effect in LM patients with EGFR mutations has not been fully evaluated. In a retrospective analysis of 51 EGFRm NSCLC patients with LM, the median LM PFS was 3.3 months (95% CI: 2.77–3.83), and no difference in LM PFS was observed between the WBRT and non‐WBRT groups (median 3.9 vs. 2.8 months; HR = 0.506, *P* = 0.052).^28^ A previous study showed that WBRT could play a role in symptom control; however, the authors did not find that it influenced the survival of LM from EGFRm NSCLC.^30^ To date, few retrospective studies have shown that WBRT could bring survival benefits to LM patients with EGFRm NSCLC, and this requires more prospective studies for further evaluation. Total spinal radiation is rarely used because of its high toxicity and a lack of evidence of survival benefit.^7^ In our study, five patients received WBRT, but no significant differences were observed in LM PFS between those who did and did not receive WBRT (6.0 vs. 9.6, *P* = 0.74). Currently, there are no standard chemotherapy regimens for NSCLC‐LM, and novel chemotherapeutic agents (such as pemetrexed combined with bevacizumab) are available. It is not known whether pemetrexed combined with bevacizumab prolongs survival in patients with LM. While intrathecal chemotherapy (ICT) has some benefit in patients with NSCLC with LM, the drug, dose, and start time of the therapy remain unclear. Methotrexate, Ara‐C, and thio‐TEPA are commonly used drugs for ICT. ICT combined with systemic chemotherapy is also not superior to single therapy, and Ommaya intracapsular administration has been reported to be superior to lumbar puncture administration.^7^ Pan *et al*. conducted a prospective single‐arm phase I clinical trial of recurrent LM and intrathecal pemetrexed for NSCLC (NCT03101579), suggesting intrathecal pemetrexed 10 mg for LM is effective with controlled toxicity. ^31^ An animal model of intrathecal bevacizumab for LM provided safety data to allow the use of bevacizumab in people with treatment‐refractory LM in phase I/II studies in the category. Although ICIs are ineffective in treating EGFRm NSCLC, there are also studies that consider ICIs after targeted treatment resistance in patients with EGFRm NSCLC. Since most clinical trials have largely excluded patients with LM, there are few data on ICIs for LM. Case reports suggest that nivolumab may have intracranial activity in patients with NSCLC CNS metastasis and good safety.^32^, ^33^ A phase II study of pembrolizumab for LM is underway (NCT03091478).

Bevacizumab is a recombinant human mAb that binds vascular endothelial growth factor‐A (VEGF‐A) and inhibits VEGFR signaling. A number of clinical trials have confirmed that VEGF inhibitors in combination with EGFR‐TKIs significantly prolong patient survival. JO25567 and NEJ026^34^ confirmed the PFS benefit of bevacizumab plus erlotinib treatment, extending PFS 3.6 months compared to erlotinib in patients with NSCLC. The ARTEMIS study was reported at the ESMO conference in 2019; the study was a phase III clinical trial of bevacizumab combined with erlotinib for the treatment of advanced EGFRm NSCLC in a Chinese population. The results showed that bevacizumab with erlotinib therapy significantly prolonged PFS (BIRC: 18.0 m vs. 11.3 m, HR = 0.55). In this study, nearly 30% of patients with baseline brain metastasis were enrolled, and PFS HR was 0.42, showing that bevacizumab with the erlotinib model may have better benefits for CNS metastasis.^35^, ^36^ Osimertinib has shown higher brain permeability than the first‐ and second‐generation treatments, and osimertinib demonstrated efficacy in NSCLC CNS metastasis. There are some studies on the treatment of TKIs in EGFRm patients with LM of NSCLC (see Table 4 for details). Overall, the median LM PFS ranged from 7.2 to 17.2 months, and the median LM OS ranged from 7.1 to 18.0 months. A 2019 ASCO Abstract (No. 9086) reported that the total remission rate of osimertinib combined with bevacizumab in patients with EGFRm lung cancer was 80%, the median PFS was 18.4 months, and osimertinib combined with bevacizumab was safe. In our study, the median LM PFS was 9.3 months, the LM ORR was 50%, and the median LM OS was 12.6 months.

**Table 4:** Studies on the treatment of TKIs in EGFRm patients with leptomeningeal metastasis (LM) of NSCLC

Time	T790M	LM patients	Median age (year)	LM PFS (months)	LM OS (months)
2018	13	20	61.2	17.2	18.0 ^37^
2019	0	5	43^38^	−	−
2016	−	184	57	−	8.9 ^39^
2020	20	41	59	8.6	11.0
2020	+	22	58	11.1	18.8 ^20^
2020	−	117	56	−	7.1 ^40^
2018	+	13	67	7.2^41^	−

EGRFm, epidermal growth factor receptor mutation; LM, leptomeningeal metastasis; OS, overall survival; NSCLC, non‐small cell lung cancer; PFS, progression‐free survival; TKIs, tyrosine kinase inhibitors.

These data, along with the findings from our study, suggest that osimertinib combined with bevacizumab is likely to be the most appropriate method to treat patients with LM from EGFRm NSCLC; however, further prospective studies are required.

Our study has some limitations. First, we included only patients from a single institution, and LM is a rare complication of NSCLC and has low prevalence; therefore, the number of patients was low, thereby explaining why only a small number of case reports and small series have been published to date in this setting.^37^, ^42^ Due to the small sample size, the present results must be interpreted cautiously. Second, the data were obtained from medical files, and we cannot exclude the possibility of undefined biases and/or confounding factors. Third, because lumbar puncture is an invasive procedure, the response criteria of MRI were judged according to the improvement of clinical symptoms and the performance of LM in our study. The limitation of PFS evaluation was that it cannot be evaluated quantitatively.

To conclude, this analysis demonstrated that osimertinib combined with bevacizumab has a clinically meaningful benefit for patients with LM from EGFRm NSCLC, suggesting that osimertinib combined with bevacizumab is an appropriate treatment option for patients with LM from EGFRm NSCLC.

## Disclosure

The authors report no conflict of interest.
